# Nutrition and Healthy Ageing in Asia: A Systematic Review

**DOI:** 10.3390/nu15143153

**Published:** 2023-07-14

**Authors:** Yan-Feng Zhou, Xing-Yue Song, An Pan, Woon-Puay Koh

**Affiliations:** 1 Department of Social Medicine, School of Public Health, Guangxi Medical University, Nanning 530021, China; 2 Department of Emergency, Hainan Clinical Research Center for Acute and Critical Diseases, The Second Affiliated Hospital of Hainan Medical University, Haikou 570311, China; songxingyue@hust.edu.cn; 3 Department of Epidemiology and Biostatistics, School of Public Health, Tongji Medical College, Huazhong University of Science and Technology, Wuhan 430032, China; panan@hust.edu.cn; 4 Healthy Longevity Translational Research Programme, Yong Loo Lin School of Medicine, National University of Singapore, Singapore 119077, Singapore; kohwp@nus.edu.sg; 5 Singapore Institute for Clinical Sciences, Agency for Science Technology and Research (A*STAR), Singapore 138632, Singapore

**Keywords:** nutrition, diet, healthy ageing, Asia, cohort

## Abstract

Background: Nutrition plays a key role in modulating the likelihood of healthy ageing. In the present study, we aimed to conduct a systematic review to assess the impact of nutrition on healthy ageing in Asia. Methods: The systematic review was registered in the International Prospective Register of Systematic Reviews database (CRD42023408936) and conducted based on the Preferred Reporting Items for Systematic Reviews and Meta-Analyses guidelines. The PubMed, Web of Science, and Embase databases were searched up to February 2023 without language restrictions. We included prospective cohort studies that evaluated the associations of intake of a single food or consumption of a single nutrient at midlife; adherence to various dietary patterns at midlife; and improved adherence to dietary patterns from mid- to late life with the likelihood of healthy ageing and its components. Results: Out of 16,373 records, we included 71 papers comprising 24 cohorts from Singapore, China, Japan, and Thailand. The healthy ageing components included cognitive function, physical function, and depression. The majority of studies supported the observation that the likelihood of healthy ageing and its components in late life was positively increased by a higher consumption of healthy foods, such as vegetables, fruits, fish, nuts, legumes, tea, milk, and dairy, at midlife, and also by greater adherence to dietary patterns with high diversity scores or high total antioxidant capacities. Furthermore, improved adherence to healthy dietary patterns from mid- to late life also increased the likelihood of healthy ageing in late life. Conclusion: Consuming healthy foods and adhering to healthy dietary patterns at midlife can promote the likelihood of healthy ageing. Moreover, improving diet quality from mid- to late life can still be beneficial.

## 1. Introduction

An increase in life expectancy and a decline in fertility rates have resulted in accelerated ageing of the population in many countries, including those in Asia. By 2050, a quarter of Asia’s population is predicted to be ≥60 years old, which will inevitably lead to an increased number of older adults with chronic diseases and disability, and with profound consequences for health, health systems, the workforce, and budgeting for many Asian countries [1]. To provide a public health framework for action, World Health Organization has released the “World report on ageing and health”, which calls for comprehensive public health action to promote healthy ageing, the latter being defined as developing and maintaining the functional ability that enables well-being in older age [2].

Nutrition and diet have been established as possessing some of the most important influences on health and ageing, with the overwhelming majority of evidence coming from Western populations [3,4]. However, there is still limited evidence on the associations between diet and nutrition at midlife and the likelihood of a multidimensional concept of healthy ageing and its components in late life in Asian populations.

In the present review, we aimed to conduct a comprehensive overview of Asian studies on the prospective associations of consumption of a single food or nutrient at midlife; adherence to various dietary patterns at midlife; and improved adherence to dietary patterns from mid- to late life with the likelihood of healthy ageing and its components. The results of this review could provide important evidence to develop better region-specific strategies aimed at promoting healthy ageing in Asia.

## 2. Methods

This systematic review was registered in the International Prospective Register of Systematic Reviews database (CRD42023408936). We followed the Preferred Reporting Items for Systematic Reviews and Meta-Analyses statement. The first two authors (Y.F.Z. and X.Y.S.) independently performed the study selection, data extraction, and assessment of study quality, and divergences were solved by discussion or consulting a third author (K.W.P.).

### 2.1. Data Sources and Searches

PubMed, EMBASE, and Web of Science were searched for studies investigating the relationship between nutrition and healthy ageing from the database’s inception to February 2023. Table S1 shows the strategies used for each database. In brief, the search terms included the Medical Subject Heading terms and related exploded versions, as well as keywords in titles or abstracts related to the following themes: ‘diet’, ‘nutrition’, ‘food’, ‘dietary pattern’, ‘healthy aging’, ‘dementia’, ‘cognitive’, ‘depression’, ‘activities of daily living’, ‘physical function’, ‘self-perceived health’, ‘ function-limiting’, ‘major chronic diseases’, ‘cohort’, ‘prospective’, ‘follow up’, and ‘longitudinal’. No language restriction was applied. In addition, reference lists of the included studies and relevant reviews were searched to identify further publications. We included cohort studies conducted in countries of Asia (defined as Eastern Asia, Southern Asia, and Southeastern Asia) and outcome measures assessed in older adults (aged ≥ 60 years). Although the global cut-off for older persons is ≥65 years, we included those aged 60–65 years as well, in order to account for a different definition of ‘older adults’ in some Asian countries [5].

### 2.2. Study Selection

The following types of studies were excluded: (1) duplicate publications or those reporting from the same cohort (the one with smaller sample size or shorter follow-up duration would be excluded); (2) unrelated to nutrition or healthy ageing; (3) not a prospective design; (4) not from a peer-reviewed publication; (5) ageing outcomes measured in those below aged <60 years; (6) and not conducted in Asia.

### 2.3. Data Extraction and Quality Assessment

Predesigned tables were used to extract information, including cohort name, country, sample size, age, median/mean follow-up duration, definition and acquisition of exposure, and assessment of outcome. The Newcastle–Ottawa Scale was used to assess the quality of the studies. A study was considered high quality if it received ≥6 points out of 9 points [6].

## 3. Results

### 3.1. Study Selection and Characteristics

**Figure 1:**
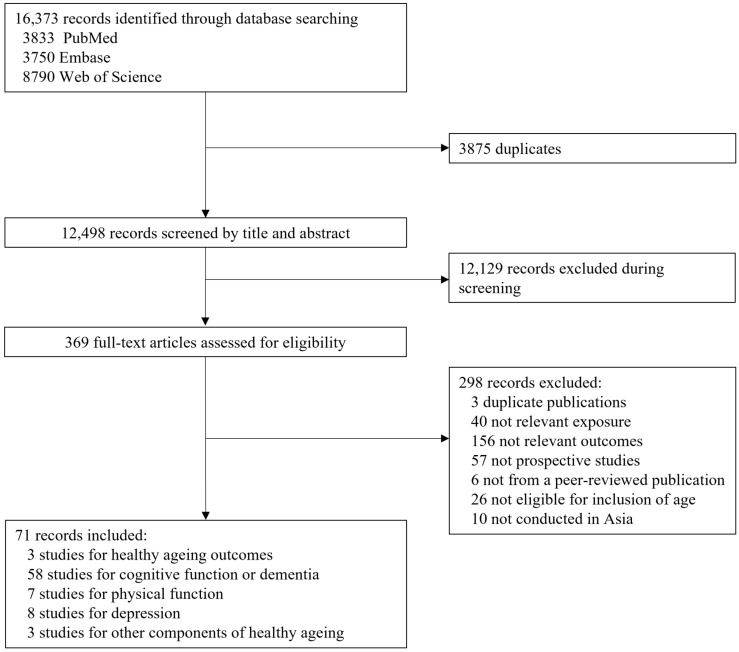
Flow chart of the study selection.

We identified 16,373 studies in the literature search. Among these, 3875 duplicates were excluded. After screening the titles and abstracts, 12,129 citations were excluded, and the remaining 369 studies were included for full-text assessment. We further excluded 298 articles after full-text reading (reasons are shown in Figure 1) and included 71 studies comprising 24 cohorts in this review (Figure 1).

The quality of the included studies, as assessed using the Newcastle–Ottawa Scale, was considered to be high for all 71 studies (Table S2). The characteristics of the eligible studies are shown in Table 1. Sixteen cohorts were from China, five from Japan, two from Singapore, and one from Thailand. Most of the studies were conducted among middle-aged or older participants, ranging in age from 40 to 89.2 years. The sample size ranged between 427 and 41,447, and the follow-up period ranged between 1.4 and 25.0 years. Food frequency questionnaires (FFQs) were used for data collection in most cohorts, except for the China Health and Nutrition Survey (CHNS), the Singapore Longitudinal Aging Studies, the National Institute for Longevity Sciences—Longitudinal Study of Aging (NILS-LSA), and the Zhejiang Ageing and Health Cohort Study. In these studies, 24 h dietary recalls for 3 consecutive days [7], 3-day dietary records [8,9,10], or simple food consumption questions [11,12,13] were used.

### 3.2. Association between Nutrition and Healthy Ageing

**Figure 2:**
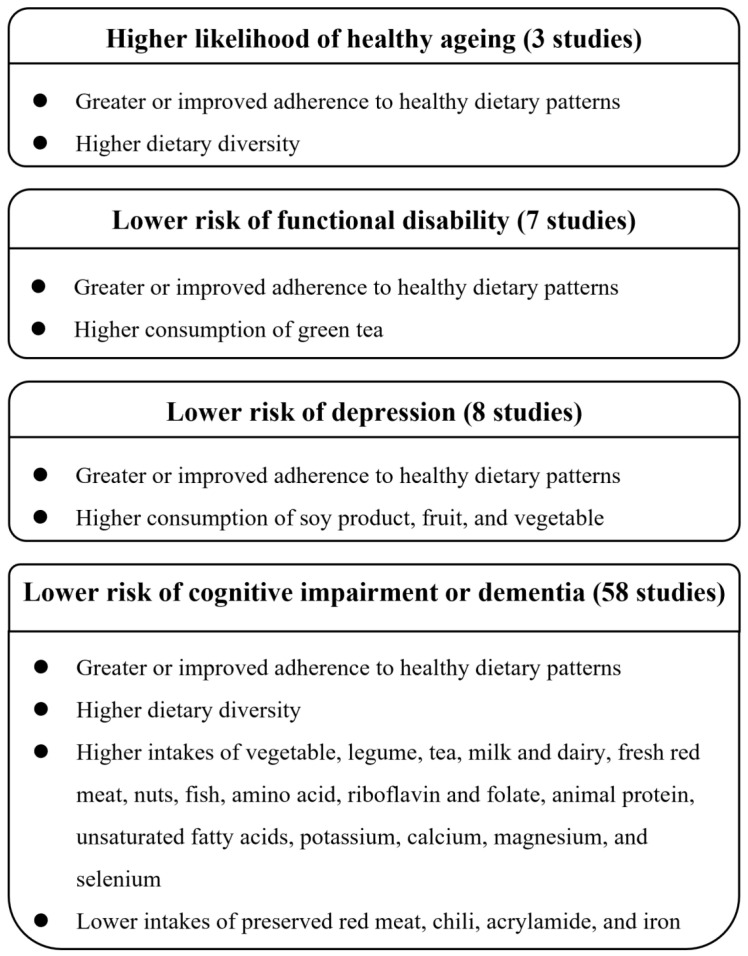
Summary of major findings regarding the associations between diet/nutrition and outcomes of ageing.

Three studies [7,14,15], which included 17,244 participants in two cohorts, investigated the multidimensional concept of healthy ageing. In the SCHS, healthy ageing was defined as the absence of specific chronic diseases; good mental and overall self-perceived health; good physical functioning; and a lack of adverse outcomes of cognitive impairment, limitations in instrumental activities of daily living (IADL), or function-limiting pain [14,15]. Data from the SCHS reported that a greater adherence to various healthy dietary patterns at midlife, defined by the alternate Mediterranean diet (aMED), the Dietary Approaches to Stop Hypertension (DASH) diet, the Alternative Healthy Eating Index (AHEI)-2010, the overall plant-based diet index (PDI), and the healthful plant-based diet index (hPDI), was associated with a higher likelihood of healthy ageing in late life, with the odds ratio (OR) comparing the highest with the lowest quartile of diet quality scores ranging from 34% to 53% for healthy ageing [14]. Furthermore, consistent or improved adherence to the DASH diet from mid- to late life was associated with a 19% to 108% higher likelihood of healthy ageing [15]. In the CHNS, a healthy ageing score was calculated by adding up the standardized scores for physical functional limitation, comorbidity, cognitive function, and psychological stress, with a lower score indicating a healthier ageing process [7]. Data from the CHNS revealed that a higher level of dietary diversity was associated with a lower score, representing healthier ageing (T3 vs. T1: β, −0.16; 95% confidence interval [CI], −0.20 to −0.11) [7]. A summary of the associations between diet/nutrition and the outcomes of ageing is presented in Figure 2.

### 3.3. Association between Nutrition and Physical Function

Seven studies [14,15,16,17,18,19,20], which included 48,674 participants, studied physical function components and how they are affected by ageing. Among these, physical function was assessed using the eight-item IADL scale [14,15,16], the Long-Term Care Insurance (LTCI) certification [17,19,20], or by the self-reported ability to conduct five self-care tasks (standing up after sitting for a long time, dressing, toileting, bathing, and feeding) [18]. Inconsistent findings were found regarding the association between the dietary diversity score and IADL limitation or incident disability, with one study showing a higher average dietary diversity score to be associated with a decreased risk of ADL disability (T3 vs. T1: hazard ratio, 0.50; 95% CI, 0.39–0.66) [18], while other studies reported null associations [16,17]. Regarding dietary patterns, greater adherence to various healthy dietary patterns [14,16,19], such as aMED, DASH, AHEI-2010, PDI, hPDI diet, fruit–egg–milk pattern, vegetable–meat–fish pattern, condiment and tea pattern, and the improved Japanese Diet Index, as well as increased adherence to the DASH diet [15], was significantly associated with a lower risk of IADL limitation or functional disability. For individual nutrients, data from the Ohsaki Cohort 2006 study showed that a higher consumption of green tea was significantly associated with a lower risk of incidents of functional disability, with a hazard ratio (95% CI) of 0.90 (0.77–1.06) among respondents who consumed 1–2 cups green tea/d; 0.75 (0.64–0.88) for those who consumed 3–4 cups/d; and 0.67 (0.57–0.79) for those who consumed ≥5 cups/d in comparison with those who consumed <1 cup/d (*p*-trend < 0.001) [20].

### 3.4. Association between Nutrition and Depression

Eight studies [13,14,15,21,22,23,24,25], which included 33,935 participants, investigated the components of depression in ageing. Among these, depression was assessed using the Center for Epidemiological Scale—Depression (CES-D) score [23,24,25], the Geriatric Depression Scale (GDS) [14,15,21], the Patient Health Questionnaire-9 (PHQ-9) [13], or the PhenX Toolkit [22]. As for dietary patterns, greater adherence to established healthy dietary patterns, such as the aMED, DASH, AHEI-2010, PDI, and hPDI diets [14], as well as an improvement in diet quality measured by these patterns [15], was associated with a lower risk of depression. However, for dietary patterns identified through a posteriori analytic methodology, while there were no significant associations of ‘vegetables-fruits’, ‘snacks-drinks-milk products’ and ‘meat-fish’ dietary patterns with a subsequent report of depressive symptoms among Chinese in Hong Kong [21], the vegetable–egg–beans–milk dietary pattern was associated with a lower risk of depression (OR, 0.65; 95% CI, 0.49–0.87), and the salt-preserved vegetable–garlic dietary pattern was associated with a higher risk of depression (OR, 1.33; 95% CI, 1.00–1.77) according to a study from the CLHLS [22]. For individual foods, higher intakes of soy products, fruits, and vegetables were associated with a lower risk of depression [13,24,25], whereas other food categories, including eggs, meat/poultry, seafood, dairy, legumes, grains, and tea, showed no significant associations [24]. Inconsistent results were shown for fish intake, with some studies reporting an inverse association [23] and others reporting null association [24].

### 3.5. Association between Nutrition and Cognitive Function or Dementia

Fifty-eight studies, which included 488,056 participants, investigated cognitive function components of ageing. Among these, cognitive function was assessed using the Telephone Interview for Cognitive Status—modified (TICS-m) [7,26,27,28,29,30,31,32,33,34,35,36,37], the Mini-Mental State Examination (MMSE) [8,9,11,12,38,39,40,41,42,43,44,45,46,47,48,49,50,51,52,59,61,65,73,77], the Montreal Cognitive Assessment (MoCA) [64,68,74,75], the Short Portable Mental Status Questionnaire (SPMSQ) [69,70], or the World Health Organization/University of California-Los Angeles Auditory Verbal Learning Test (AVLT) [71], or was evaluated by asking questions about walking capability, hearing/vision, memory, and decision-making ability [72]. Diagnoses of dementia were made in accordance with the Diagnostic and Statistical Manual of Mental Disorders [53,54,55,56,60,76]; the criteria of the LTCI certification [10,57,58,62,63]; or the International Statistical Classification of Diseases and Related Health Problems, Tenth Revision (ICD-10) [67]; or were obtained from the National Health Insurance Database [66].

For dietary diversity, a higher score was associated with a lower risk of cognitive impairment [7,38], bad memory [26], and disabling dementia [57]. Regarding dietary patterns, greater adherence to healthy dietary patterns, such as the aMED, DASH, AHEI-2010, modified AHEI, PDI, hPDI diet [39,40,43,64,71], Chinese Food Pagoda [72], “vegetable” [68] or “vegetable-pork” dietary pattern [30], “protein-rich” dietary pattern [29], beans and mushroom dietary pattern [28], Japanese dietary pattern [10,53,63], or wheat-based diverse diet [27], as well as improvements in diet quality [44], were associated with a lower risk of cognitive impairment, cognitive/memory decline, and incident dementia. However, the Western dietary pattern [69], animal-based dietary pattern [41], unhealthful PDI [39], and starch-rich dietary pattern [29] increased the risk of cognitive decline.

For individual foods, higher intakes of vegetables and their constituent nutrients [50,54,64,65,67,70], legumes [42], tea [8,34,66], milk and dairy [48,55], fresh red meat [47,74], nuts [31,51], and fish [32,60,62,66] were associated with a lower risk of cognitive impairment, cognitive decline, and dementia. For individual nutrients, a higher dietary total antioxidant capacity [45] and higher intakes of amino acids [61], riboflavin and folate [49,75], animal protein [33], unsaturated fatty acids, polyunsaturated fatty acids (PUFAs), and n-3 PUFA supplements [11,52,60,75] were associated with a lower risk of cognitive impairment, cognitive decline, and dementia. In contrast, higher consumption of preserved red meat [47], chili [35], acrylamide [73], and protein intake from grains [33] were associated with a higher risk of cognitive impairment. No significant associations were found for thiamine, niacin, vitamin B-6 [49], sugar-sweetened beverages [46], or coffee [8]. Inconsistent findings were found for intakes of fruit, soy, and isoflavones, as well as vitamin B-12, with different studies showing either inverse associations [9,50,67,70], positive associations [59], or a U-shaped relation [77], and others reporting null associations [48,49,54,58,65].

For dietary minerals, higher intakes of potassium, calcium, magnesium, and selenium were associated with a lower risk of dementia [54,56,76] or a reduced likelihood of reporting memory decline [37], whereas a higher iron intake was associated with poorer cognitive function [36].

### 3.6. Association between Nutrition and Other Components of Healthy Ageing

For other components of healthy ageing, greater adherence to various healthy dietary patterns at midlife, as well as consistent or improved adherence to the DASH diet from mid- to late life, was associated with a higher likelihood of having good self-perceived health and physical functioning and a lower likelihood of having chronic diseases and function-limiting pain [14,15]. In addition, a higher dietary diversity score was associated with less psychological stress (T3 vs. T1: OR, 0.59; 95% CI, 0.49–0.72); however, the association between the dietary diversity score and the number of comorbidities was insignificant [7].

## 4. Discussion

In this systematic review, we used data from population-based longitudinal cohort studies to investigate the prospective associations between nutrition at midlife and the likelihood of healthy ageing and its components in late life in Asia. Most of the current evidence has supported the positive associations of higher intakes of healthy foods at midlife, such as vegetables, fruits, fish, nuts, legumes, tea, milk, and dairy. Furthermore, a higher dietary diversity or total dietary antioxidant capacity at midlife, as well as greater or improved adherence to healthy dietary patterns from mid- to late life, was also associated with the likelihood of healthy ageing and its components in late life.

The currently available literature supports that adherence to various healthy dietary patterns is associated with a higher likelihood of healthy ageing. These healthy dietary patterns, either determined a priori or identified through a posteriori analytic methodology, are similar in that they recommend high consumption of fruits, vegetables, and whole grains; moderate consumption of dairy products, fish, and poultry; and low consumption of sugary beverages, saturated fat, added sodium, red meat, and processed food [14,16,19,39,40,43,54,64,71]. However, these results should be interpreted with caution, given that differences exist in the major ingredients and culinary methods used between Asian and Western cuisines. For example, the Mediterranean diet emphasizes fruits, vegetables, whole grains, and olive oil as staples, while Asian diets commonly rely on white rice, noodles, and other grains as primary sources of energy [72]. This variation in staple foods may significantly impact nutrient composition and overall dietary patterns.

In addition, the findings confirmed that maintaining consistently high DASH scores was related to a greater likelihood of healthy ageing than keeping consistently low DASH scores [15]. Moreover, those who managed to improve their DASH scores by >10% from mid- to late life were able to increase their likelihood of healthy ageing [15]. Hence, our findings provide evidence for the recommendation of the 2020–2025 Dietary Guidelines Advisory Committee that “it is never too late to eat healthfully” [78]. More studies are warranted to explore strategies in order to achieve a sustained change in dietary behaviours in the real world and to create an environment in which to make healthy eating affordable and accessible.

Dietary diversity is an important index reflecting nutrient adequacy. Increasing dietary diversity can ensure sufficient nutrient intake and improve dietary quality to promote healthy ageing [7,38]. However, mixed findings were observed regarding the association between dietary diversity score and IADL limitation or incident disability. Data from the CLHLS, including 2285 subjects aged >60 years with a maximum follow-up of 7 years, reported that dietary diversity had no effect on the occurrence of IADL limitation [16]. The Ota Genki Senior Project, including 10,318 Japanese adults aged >65 years with a median follow-up of 5.1 years, found that dietary variety was not independently associated with incident disability [17]. However, data from 5004 participants in a study of the CHNS reported that higher dietary diversity scores were associated with fewer physical functional limitations [18]. There are several potential reasons for these mixed findings. First, there is substantial variability in the measures of physical function and functional disability due to the use of different scales and instruments in different studies. Second, the intake frequency and scoring criteria of dietary diversity scores varied substantially across studies. For example, the dietary diversity score was calculated according to the intake frequency of 13 food groups, and the low group was defined as <7 in the CLHLS [16], whereas it was calculated according to the intake frequency of 10 food groups and a low group was defined as <3 in the Ota Genki Senior Project [17]. Nevertheless, our review concurs with the World Health Organization [79] and Chinese dietary guidelines [80] in terms of recommending adherence to a diverse diet to achieve healthy ageing in later life.

The associations between the intakes of fruits and fish and the likelihood of healthy aging components were inconsistent, and this could be explained by differences in the ranges of consumption among different populations. For example, the Hisayama study, which included 1071 Japanese participants, observed small differences among quintiles of fruit intake, with the range of the highest quartile of fruit intake being ≥115 g/d for men (≥100 g/d for women) and the lowest quartile being ≤32 g/d for men (≤21 g/d for women) [54]. However, there were substantial differences among the quartiles of fruit intake in the SCHS, with the median fruit intake in the highest and lowest quartile being 383.44 g/d and 76.30 g/d, respectively [50]. Notably, the SCHS applied a 165-item FFQ which included 14 fruits [50], whereas the Hisayama study applied a 70-item FFQ, and might have underestimated the fruit intake in this population [54]. Differences in methods of categorizing the intake of fish across studies could also explain these inconsistent results. For example, fish intake was divided into <3 times/week and ≥3 times/week in the Survey of Health and Living Status of the Elderly, and a null effect was reported for fish intake and risk of depression [24]. In contrast, fish intake was divided according to quartile consumption in the JPHC study, and a reduced risk of major depressive disorder was found in the third quartile (111.1 g/d) [23].

To the best of our knowledge, this is the first study which has systematically reviewed the association between nutrition in midlife and the likelihood of healthy ageing in late life according to Asian cohort studies. In addition, the quality of the included studies was considered to be high. Several limitations should be considered. First, except for the analyses of the association between nutrition and cognitive function, analyses related to healthy ageing, physical function, depression, and other components of healthy ageing only included limited studies. In addition, although we included 71 studies from 24 cohorts, these cohorts were situated in China, Japan, Singapore and Thailand, and represented a small proportion of the diverse Asian population. Second, substantial variations existed across the studies in terms of the measures of exposure, definitions of outcomes, sample sizes, and follow-up durations. Nonetheless, the overall results are consistent in that they recommend the consumption of healthy foods and adherence to healthy dietary patterns at midlife for healthy ageing. Moreover, improving the quality of one’s diet from mid- to late life can still be beneficial.

## 5. Conclusions

The present study identified associations between nutrition at midlife and the likelihood of healthy ageing in late life using robust data from cohort studies in Asia. Our study’s results provide important evidence for policymaking and dietary guidelines aimed at promoting healthy ageing in Asia.

## Figures and Tables

**Table 1:** Characteristics of studies included in the systematic review.

Author, Year (Ref)	Cohort	Country	Participants, n	Age, y *	Follow-Up, y *	Nutrition	Nutrition Measures	Ageing Outcomes	Outcome Definition or Measures
Zhang et al., 2021 [7]	The CHNS	China	3085	>60	4–6	Dietary diversity score	24 h dietary recalls for 3 consecutive days	Healthy ageing, physical and cognitive function	Healthy ageing included physiological, psychological, and sociological aspects.
Zhou et al., 2020 [14]	The Singapore Chinese Health Study (SCHS)	Singapore	14,159	53.3 (6.1)	20	Diet quality, scored by the aMED, DASH diet, AHEI-2010, PDI, and hPDI.	165 items, semi-quantitative FFQ	Healthy ageing, depression, physical function	Healthy ageing included: major chronic diseases, cognitive function, IADL, depression, self-perceived health, physical functioning, and function-limiting pain.
Zhou et al., 2022 [15]	The SCHS	Singapore	12,316	53.1	20	Changes in DASH scores	165 items, semi-quantitative FFQ	Healthy ageing, depression, physical function	Healthy ageing included: major chronic diseases, cognitive function, IADL, depression, self-perceived health, physical functioning, and function-limiting pain.
Aihemaitijiang et al., 2022 [16]	The Chinese Longitudinal Healthy Longevity Survey (CLHLS)	China	2282	≥60	7	Dietary diversity score	A non-quantitative frequency questionnaire of 13 food groups	Physical function	Physical function was judged according to the 8-item IADL.
Hata et al., 2022 [17]	The Ota Genki Senior Project	Japan	10,318	≥65	5.1	Dietary variety score	A 10-item FFQ	Physical function	Functional disability was defined by the LTCI certification.
Zhang et al., 2020 [18]	The CHNS	China	5004	58.6	9	Dietary diversity score	24 h dietary recalls for 3 consecutive days	Physical function	ADL disability was defined as having any difficulty in at least one of the five self-care tasks.
Matsuyama et al. 2019 [19]	The Ohsaki Cohort 2006 Study	Japan	2923	≥65	10	The Japanese Diet Index	A 39-item FFQ	Physical function	Functional disability was defined using the LTCI certification.
Tomata et al., 2012 [20]	The Ohsaki Cohort 2006 Study	Japan	13,988	≥65	3	Green tea	A 39-item FFQ	Physical function	Functional disability was defined using the LTCI certification.
Chan et al., 2014 [21]	The Mr. and Ms. Os cohort	China, Hong Kong	4000	≥65	3.9	Dietary patterns related to vegetables, fruits, snacks, drinks, milk products, and meat/fish	A 280-item FFQ	Depression	Depression was assessed usingusing the GDS.
Pei et al., 2022 [22]	The CLHLS	China	2873	80.3	4	Dietary patterns	A non-quantitative frequency questionnaire of 13 food groups	Depression	Depression was assessed usingusing the PhenX Toolkit
Matsuoka et al., 2017 [23]	The Japan Public Health Center-based Prospective (JPHC) Study	Japan	1181	40–69	Up to 25	Fish intake and PUFA	A 147-item FFQ	Depression	Depression was assessed usingusing the CES-D.
Tsai et al., 2011 [24]	The Survey of Health and Living Status of the Elderly in Taiwan	China	1069	≥65	4	Vegetables and fruits	An FFQ covering 7 food categories	Depression	Depression was assessed usingusing the CES-D.
Fann et al., 2022 [25]	The Taiwan Longitudinal Survey on Aging (TLSA)	China	4400	≥53	16	Vegetables and fruits	An FFQ covering 9 food categories	Depression	Depression was assessed usingusing the CES-D.
Zhang et al., 2022 [13]	The Zhejiang Ageing and Health Cohort Study	China	6253	68.2	6	Soy products	Single question	Depression	Depression was assessed usingusing the PHQ-9.
Zhang et al., 2020 [26]	The CHNS	China	4356	61.9 (7.9)	4	Dietary diversity score	24 h dietary recalls for 3 consecutive days	Cognitive function	Cognitive function was assessed usingusing the TICS-m.
Qin et al., 2015 [27]	The CHNS	China	1650	≥55	5.3	Dietary pattern, aMED	24 h dietary recalls for 3 consecutive days	Cognitive function	Cognitive function was assessed usingusing the TICS-m.
Shang et al., 2021 [28]	The CHNS	China	2307	63.3 (7.0)	7 (2–11)	Five dietary patterns	24 h dietary recalls for 3 consecutive days	Cognitive function	Cognitive function was assessed usingusing the TICS-m.
Xu et al., 2018 [29]	The CHNS	China	4874	64 (59, 71)	-	Dietary patterns: traditional Chinese, protein-rich, starch-rich	24 h dietary recalls for 3 consecutive days	Cognitive function	Cognitive function was assessed usingusing the TICS-m.
Zhang et al., 2023 [30]	The CHNS	China	6308	≥55	Up to 21	A vegetable-pork dietary pattern	24 h dietary recalls for 3 consecutive days	Cognitive function	Cognitive function was assessed usingusing the TICS-m.
Li et al., 2019 [31]	The CHNS	China	4822	≥55	15	Nut intake	24 h dietary recalls for 3 consecutive days	Cognitive function	Cognitive function was assessed usingusing the TICS-m.
Qin et al., 2014 [32]	The CHNS	China	1566	63 (6)	5.3	Fish intake	24 h dietary recalls for 3 consecutive days	Cognitive function	Cognitive function was assessed usingusing the TICS-m.
Gao et al., 2022 [33]	The CHNS	China	3083	61.9 (6.6)	9 (2–18)	Protein intake from grains	24 h dietary recalls for 3 consecutive days	Cognitive function	Cognitive function was assessed usingusing the TICS-m.
Sukik et al., 2022 [34]	The CHNS	China	4657	62.8	Up to 14	Tea consumption	24 h dietary recalls for 3 consecutive days	Cognitive function	Cognitive function was assessed usingusing the TICS-m.
Shi et al., 2019 [35]	The CHNS	China	4852	63.4 (7.7)	Up to 15	Chili Intake	24 h dietary recalls for 3 consecutive days	Cognitive function	Cognitive function was assessed usingusing the TICS-m.
Shi et al., 2019 [36]	The CHNS	China	4852	63.4 (7.7)	Up to 15	Iron intake	24 h dietary recalls for 3 consecutive days	Cognitive function	Cognitive function was assessed usingusing the TICS-m.
Jiang et al., 2022 [37]	The CHNS	China	4852	63.4 (7.7)	Up to 15	Selenium intake	24 h dietary recalls for 3 consecutive days	Cognitive function	Cognitive function was assessed usingusing the TICS-m.
Zheng et al., 2021 [38]	The CLHLS	China	11,970	89.2 (6.9)	3.9 (1.4–16.4)	Dietary diversity score	A non-quantitative frequency questionnaire of 13 food groups	Cognitive function	Cognitive function was assessed usingusing the MMSE.
Zhu et al., 2022 [39]	The CLHLS	China	6136	80.0 (9.8)	10	Dietary pattern, PDI, hPDI, uPDI	A non-quantitative frequency questionnaire of 13 food groups	Cognitive function	Cognitive function was assessed usingusing the MMSE.
Wang et al., 2020 [40]	The CLHLS	China	5716	82	3	A healthy dietary pattern of eight food groups	A non-quantitative frequency questionnaire of 13 food groups	Cognitive function	Cognitive function was assessed usingusing the MMSE.
Hu et al., 2023 [41]	The CLHLS	China	17,827	86.3 (10.2)	-	The animal-based diet index	A non-quantitative frequency questionnaire of 13 food groups	Cognitive function	Cognitive function was assessed usingusing the MMSE.
Chen et al., 2012 [42]	The CLHLS	China	5691	89.2 (10.1)	3	Vegetables and legumes	A non-quantitative frequency questionnaire of 13 food groups	Cognitive function	Cognitive function was assessed using the MMSE.
Wu et al., 2019 [43]	The SCHS	Singapore	16,948	53.5 (6.2)	20.2 (1.9)	Dietary patterns, aMED, DASH, AHEI- 2010, PDI, hPDI	A 165-item semi-quantitative FFQ	Cognitive function	Cognitive function was assessed using the MMSE.
Tong et al., 2021 [44]	The SCHS	Singapore	14,683	53.5 (6.2)	19.7	Changes in DASH score	A 165-item semi-quantitative FFQ	Cognitive function	Cognitive function was assessed using the MMSE.
Sheng et al., 2021 [45]	The SCHS	Singapore	16,703	53.5 (6.2)	20.2 (1.9)	Total antioxidant capacity	A 165-item semi-quantitative FFQ	Cognitive function	Cognitive function was assessed using the MMSE.
Zhang et al., 2020 [46]	The SCHS	Singapore	16,948	53.5 (6.2)	20.2 (1.9)	Sugar-sweetened beverages consumption	A 165-item semi-quantitative FFQ	Cognitive function	Cognitive function was assessed using the MMSE.
Jiang et al., 2020 [47]	The SCHS	Singapore	16,948	53.5 (6.2)	20.2 (1.9)	Meat intake	A 165-item semi-quantitative FFQ	Cognitive function	Cognitive function was assessed using the MMSE.
Talaei et al., 2021 [48]	The SCHS	Singapore	16,948	53.5 (6.2)	20.2 (1.9)	Dairy, soy, and calcium consumption	A 165-item semi-quantitative FFQ	Cognitive function	Cognitive function was assessed using the MMSE.
Sheng et al., 2020 [49]	The SCHS	Singapore	16,948	53.5 (6.2)	20.2 (1.9)	B vitamins intake	A 165-item semi-quantitative FFQ	Cognitive function	Cognitive function was assessed using the MMSE.
Sheng et al., 2022 [50]	The SCHS	Singapore	16,737	53.5 (6.2)	20.2 (1.9)	Fruit and vegetable intake	A 165-item semi-quantitative FFQ	Cognitive function	Cognitive function was assessed using the MMSE.
Jiang et al., 2021 [51]	The SCHS	Singapore	16,737	53.5 (6.2)	20.2 (1.9)	Nut intake	A 165-item semi-quantitative FFQ	Cognitive function	Cognitive function was assessed using the MMSE.
Jiang et al., 2020 [52]	The SCHS	Singapore	16,736	53.5 (6.2)	20.2 (1.9)	Monounsaturated acids, n–6 Polyunsaturated acids, and Plant-based fat intake	A 165-item semi-quantitative FFQ	Cognitive function	Cognitive function was assessed using the MMSE.
Ozawa et al., 2013 [53]	The Hisayama study	Japan	1006	68	15	Dietary pattern	A 70-item semiquantitative FFQ	Dementia	Diagnosis of dementia was made in accordance with the Diagnostic and Statistical Manual of Mental Disorders.
Kimura al, 2022 [54]	The Hisayama study	Japan	1071	≥60	Up to 24	Vegetable and fruit intake	A 70-item semiquantitative FFQ	Dementia	Diagnosis of dementia was made in accordance with the Diagnostic and Statistical Manual of Mental Disorders.
Ozawa et al., 2014 [55]	The Hisayama study	Japan	1081	≥60	17	Milk and dairy consumption	A 70-item semiquantitative FFQ	Dementia	Diagnosis of dementia was made in accordance with the Diagnostic and Statistical Manual of Mental Disorders.
Ozawa et al., 2012 [56]	The Hisayama study	Japan	1081	≥60	17	Potassium, calcium, and magnesium Intake	A 70-item semiquantitative FFQ	Dementia	Diagnosis of dementia was made in accordance with the Diagnostic and Statistical Manual of Mental Disorders.
Otsuka et al., 2023 [57]	The JPHC Study	Japan	38,797	45–74	11	Dietary diversity score	A self-administered 147-item FFQ	Dementia	Dementia was made in accordance with the LTCI certification
Murai et al., 2021 [58]	The JPHC Study	Japan	41,447	45–74	9.4	Soy product intake	A self-administered 147-item FFQ	Dementia	Dementia was made in accordance with the LTCI certification
Svensson et al., 2022 [59]	The JPHC Saku Mental Health Study	Japan	1036	40–59	-	Soy and isoflavone intake	A self-administered 147-item FFQ	Dementia	Dementia was determined in accordance with the LTCI certification.
Nozakia et al., 2021 [60]	The JPHC Saku Mental Health Study	Japan	1127	45–64	Up to 20	Fish and n-3 polyunsaturated fatty acid (PUFA) consumption	A self-administered 147-item FFQ	Dementia	Dementia was determined in accordance with the LTCI certification.
Zhang et al., 2023 [10]	The NILS-LSA	Japan	1504	65–82	11.4	Japanese Diet Index score	3-day dietary records (3DRs)	Dementia	Dementia was determined in accordance with the LTCI certification.
Kinoshita et al., 2021 [61]	The NILS-LSA	Japan	427	67.1 (5.2)	8.2 (0.3)	Lysine, phenylalanine, threonine, and alanine intake	3-day dietary records (3DRs)	Cognitive function	Cognitive function was assessed using the MMSE.
Shirai, et al., 2019 [8]	The NILS-LSA	Japan	1305	60–85	5.3 (2.9)	Green tea and coffee intake	3-day dietary records (3DRs)	Cognitive function	Cognitive function was assessed using the MMSE.
Nakamoto et al., 2017 [9]	The NILS-LSA	Japan	776	60–81	8	Bean, soy product, and soy isoflavone intake	3-day dietary records (3DRs)	Cognitive function	Cognitive function was assessed using the MMSE.
Tsurumaki et al., 2019 [62]	The Ohsaki Cohort 2006 Study	Japan	13,102	≥65	5.7	Fish and other foods	A 39-item FFQ	Dementia	Dementia was determined in accordance with the LTCI certification.
Tomata et al., 2016 [63]	The Ohsaki Cohort 2006 Study	Japan	14,402	73.8 (5.9)	4.9 (1.5)	Three dietary patterns: Japanese pattern, animal food pattern, and high-dairy pattern.	A 39-item FFQ	Dementia	Dementia was determined in accordance with the LTCI certification.
Chou, et al., 2019 [64]	The Taiwan Initiative for Geriatric Epidemiological Research	China	436	72.5 (5.2)	2	Diet, diet quality (mAHEI), and vegetable variety	A 44-item semi-quantitative FFQ	Cognitive function	Cognitive function was assessed using the MoCA.
Li et al., 2022 [12]	The Zhejiang Ageing and Health Cohort Study	China	9028	68.7 (7.0)	6	Eggs consumption	Frequency and quantity of egg consumption intake were investigated	Cognitive function	Cognitive function was assessed using the MMSE.
Yeung, et al., 2022 [65]	The Mr. and Ms. Os cohort	China	1518	≥65	4	Fruit and vegetable intake	A validated 280-item FFQ	Cognitive function	Cognitive function was assessed using the MMSE.
Chuang et al., 2019 [66]	The Nutrition and Health Survey in Taiwan	China	1436	≥65	11.04	Consumption of tea and fish	A 79-item food frequency questionnaire	Dementia	Dementia was determined in accordance with the National Health Insurance Database.
Lee et al., 2017 [67]	A cohort study in the Elderly Health Centers in Hong Kong	China	17,700	≥65	6	Vegetable and fruit consumption	An FFQ	Dementia	Dementia was determined in accordance with the ICD-10
Chen et al., 2017 [68]	A prospective cohort study in National Taiwan University Hospital	China	475	≥65	2	Dietary pattern	A 44-item semi-quantitative FFQ	Cognitive function	Cognitive function was assessed using the MoCA.
Tsai et al., 2014 [69]	The TLSA	China	2988	73 (6)	3–4	Dietary patterns	A questionnaire on FFQ covering 9 food categories	Cognitive function	Cognitive function was assessed using the SPMSQ
Wang et al., 2022 [70]	The TLSA	China	1491	≥53	16	Fruit and vegetable intake	A questionnaire on FFQ covering 9 food categories	Cognitive function	Cognitive function was assessed using the SPMSQ
Jia et al., 2023 [71]	The China Cognition and Ageing Study	China	29,072	≥60	10	A healthy diet	A 12-item FFQ	Cognitive function	Cognitive function was assessed using the World Health Organization/University of California Los Angeles Auditory Verbal Learning Test
Zhu et al., 2018 [72]	The Shanghai Women’s Health Study and Shanghai Men’s Health Study	China	30,484	40–74	14.4	Dietary patterns, DASH, AHEI, CHFP	A 77-item FFQ	Cognitive function	Cognitive function was evaluated by asking questions about walking capability, hearing/vision, memory, and decision-making ability
Liu et al., 2017 [73]	A cohort study in the School of Public Health of the Chinese University of Hong Kong	China	2534	≥65	4	Acrylamide intake	A 329-item FFQ	Cognitive function	Cognitive function was assessed using the MMSE.
Gao et al., 2011 [11]	The Singapore Longitudinal Aging Studies	Singapore	1475	66.0	1.57	Omega-3 PUFA intake	Self-reported; a single question was asked	Cognitive function	Cognitive function was assessed using the MMSE.
Manacharoen et al., 2023 [74]	The Electricity Generating Authority of Thailand study	Thailand	821	60.0 (4.3)	5	Nine major food groups	A 40-item FFQ	Cognitive function	Cognitive function was assessed using the MoCA.
Tao et al., 2019 [75]	The Shanghai Aging Study	China	1385	58.75	2	Riboflavin and unsaturated fatty acid	An 85-item FFQ	Cognitive function	Cognitive function was assessed using the MoCA.
Luo et al., 2022 [76]	A longitudinal study in China	China	1565	71.1	5.2	Ca, Mg intake	A 111-item interviewer-administered FFQ	Dementia	Dementia was determined in accordance with the Diagnostic and Statistical Manual of Mental Disorders.
Wang et al., 2021 [77]	The Effects and Mechanism Investigation of Cholesterol and Oxysterol on Alzheimer’s disease study	China	2546	≥50	2	Four nutrient patterns	A 33-item FFQ	Cognitive function	Cognitive function was assessed using the MMSE.

AHEI, Alternative Healthy Eating Index; aMED, Alternate Mediterranean Diet score; CES-D, the Center for Epidemiological Scale—Depression; CHFP, the Chinese Food Pagoda; CHNS, China Health and Nutrition Survey; CLHLS, Chinese Longitudinal Healthy Longevity Survey; DASH, Dietary Approaches to Stop Hypertension; FFQ, food frequency questionnaire; GDS, Geriatric Depression Scale; hPDI, healthful plant-based diet index; IADL, instrumental activities of daily living; JPHC, Japan Public Health Center-based; LTCI, Long-Term Care Insurance; MMSE, Mini-Mental State Examination; MoCA, Montreal Cognitive Assessment; NILS-LSA, National Institute for Longevity Sciences—Longitudinal Study of Aging; PDI, plant-based diet index; PHQ-9, Patient Health Questionnaire-9; SCHS, Singapore Chinese Health Study; SPMSQ, Short Portable Mental Status Questionnaire; TICS-m, Telephone Interview for Cognitive Status—modified; TLSA, Taiwan Longitudinal Survey on Aging; uPDI, unhealthful plant-based diet index. * Values are ranges or means/median (standard deviation).

## Data Availability

Not applicable.

## Supplementary Materials

The following supporting information can be downloaded at: https://www.mdpi.com/article/10.3390/nu15143153/s1, Table S1: Search strategy; Table S2: Risk of bias of the included studies: the Newcastle–Ottawa Scale.
