# Supplementary material for: Nutrition and Healthy Ageing in Asia: A Systematic Review
Source: Nutrients. 2023 Jul 14;15(14):3153. doi: 10.3390/nu15143153 (PMC10383719; doi:10.3390/nu15143153)
Supplement: Supplementary file 1 [file nutrients-15-03153-s001.zip › nutrients-2479625-supplementary.pdf]

Table S1. Search strategy.

For PubMed

Number	Query
7	Search (#1 AND #2 AND #3 AND #4)
6	Search (#1 AND #2 AND #3)
5	Search (#1 AND #2)
4	Search (China[Mesh] OR Taiwan[Mesh] OR Japan[Mesh] OR Korea[Mesh] OR Mongolia[Mesh] OR Brunei[Mesh] OR Cambodia[Mesh] OR Indochina[Mesh] OR Indonesia[Mesh] OR Laos[Mesh] OR Malaysia[Mesh] OR Mekong Valley[Mesh] OR Myanmar[Mesh] OR Philippines[Mesh] OR Singapore[Mesh] OR Thailand[Mesh] OR Timor-Leste[Mesh] OR Vietnam[Mesh] OR Afghanistan[Mesh] OR Sri Lanka[Mesh] OR Bangladesh[Mesh] OR Bhutan[Mesh] OR India[Mesh] OR Nepal[Mesh] OR Pakistan[Mesh] OR Maldives[Mesh])
3	Search (cohort studies[Mesh] OR incidence[Mesh] OR survival analysis[Mesh] OR prospective[Title/Abstract] OR prospectively[Title/Abstract] OR cohort[Title/Abstract] OR follow up[Title/Abstract] OR followed up[Title/Abstract] OR longitudinal[Title/Abstract] OR incidence[Title/Abstract])
2	Search (healthy aging[Mesh] OR dementia[Mesh] OR Alzheimer Disease[Mesh] OR Cognitive Dysfunction[Mesh] OR Cognition[Mesh] OR Quality of Life[Mesh] OR Depression[Mesh] OR Activities of Daily Living[Mesh] OR aging[Title/Abstract] OR ageing[Title/Abstract] OR dementia[Title/Abstract] OR Alzheimer Disease[Title/Abstract] OR Cognit*[Title/Abstract] OR Neurocognit*[Title/Abstract] OR Quality of Life[Title/Abstract] OR Health status [Title/Abstract] OR Depression[Title/Abstract] OR Activities of Daily Living[Title/Abstract] OR physical function[Title/Abstract] OR self-perceived health[Title/Abstract] OR function-limiting[Title/Abstract] OR Cardiovascular diseases[Title/Abstract] OR stroke[Title/Abstract] OR myocardial infarction[Title/Abstract] OR heart failure[Title/Abstract] OR Cancer[Title/Abstract] OR diabetes[Title/Abstract] OR kidney[Title/Abstract] OR lung disease[Title/Abstract])
1	Search (Diet[Mesh] OR nutrition[Title/Abstract] OR food combination*[Title/Abstract] OR diet pattern*[Title/Abstract] OR dietary pattern*[Title/Abstract] OR eating pattern*[Title/Abstract] OR nutrient pattern*[Title/Abstract] OR food pattern*[Title/Abstract] OR diet habit*[Title/Abstract] OR dietary habit*[Title/Abstract] OR eating habit* [Title/Abstract] OR nutrient habit* [Title/Abstract] OR food habit* [Title/Abstract] OR vitamin* [Title/Abstract] OR diet score* [Title/Abstract] OR dietary score* [Title/Abstract] OR diet quality[Title/Abstract] OR dietary quality[Title/Abstract] OR diet index*[Title/Abstract] OR dietary index*[Title/Abstract] OR Mediterranean diet[Title/Abstract] OR Healthy Eating Index*[Title/Abstract] OR Dietary Approaches to Stop Hypertension[Title/Abstract] OR Prudent pattern* [Title/Abstract] OR conservative pattern* [Title/Abstract] OR traditional pattern* [Title/Abstract] OR vegetarian diet* [Title/Abstract] OR DASH[Title/Abstract])

For EMBASE

Number	Query
#1	exp diet/
#2	(food combination or diet* pattern or eating pattern or nutrient pattern or food pattern or diet* habit* or eating habit* or nutrient habit* or food habit* or diet* score or diet* quality or diet* index* or Mediterranean diet* or Healthy Eating Index* or Dietary Approaches to Stop Hypertension or Prudent pattern* or conservative pattern* or traditional pattern* or vegetarian diet* or DASH).ti,ab.

#3	exp healthy ageing/
#4	(healthy ag* or success ag*).ti,ab.
#5	exp dementia/ or exp cognitive defect/ or exp quality of life/ or exp depression/ or exp activities of daily living/
#6	(cognit* or neurocognit* or dementia or Alzheimer* or health* or physical function or self-perceived health or function-limiting or cardiovascular* or stroke or myocardial infarction or heart failure or cancer or diabetes or kidney or lung disease).ti,ab.
#7	exp prospective study/ or exp cohort analysis/ or exp survival analysis/ or exp follow up/ or exp longitudinal study/ or exp incidence/
#8	(cohort or incidence or survival or prospective or prospectively or follow-up or follow* up or longitudinal).ti,ab.
#9	exp China/ or exp Taiwan/ or exp Japan/ or exp Korea/ or exp Mongolia/ or exp Brunei/ or exp Cambodia/ or exp Indochina/ or exp Indonesia/ or exp Laos/ or exp Malaysia/ or exp Mekong Valley/ or exp Myanmar/ or exp Philippines/ or exp Singapore/ or exp Thailand/ or exp Timor-Leste/ or exp Vietnam/ or exp Afghanistan/ or exp Sri Lanka/ or exp Bangladesh/ or exp Bhutan/ or exp India/ or exp Nepal/ or exp Pakistan/ or exp Maldives/
#10	(China or Taiwan or Japan or Korea or Mongolia or Brunei or Cambodia or Indochina or Indonesia or Laos or Malaysia or Mekong Valley or Myanmar or Philippines or Singapore or Thailand or Timor-Leste or Vietnam or Afghanistan or Sri Lanka or Bangladesh or Bhutan or India or Nepal or Pakistan or Maldives).ti,ab.
#11	#1 OR #2
#12	#3 OR #4 OR #5 OR #6
#13	#7 OR #8
#14	#9 OR #10
#15	#11 AND #12 AND #13 AND #14

For Web of Science

Number	Query
#1	TS=(diet OR nutrition OR food combination OR Vitamins OR diet* pattern OR eating pattern OR nutrient pattern OR food pattern OR diet* habit* OR eating habit* OR nutrient habit* OR food habit* OR diet* score OR diet* quality OR diet* index* OR Mediterranean diet* OR Healthy Eating Index* OR Dietary Approaches to Stop Hypertension OR Prudent pattern* OR conservative pattern* OR traditional pattern* OR vegetarian diet* OR DASH) Indexes=SCI-EXPANDED, SSCI, A&HCI, CPCI-S, CPCI-SSH, ESCI Timespan=All years
#2	TS=(healthy aging OR dementia OR Alzheimer* OR cognit* OR neurocognit* OR Quality of Life OR Depression OR Activities of Daily Living OR ageing OR Health status OR physical function OR self-perceived health OR function-limiting OR Cardiovascular diseases OR stroke OR myocardial infarction OR heart failure OR Cancer OR diabetes OR kidney OR lung disease) Indexes=SCI-EXPANDED, SSCI, A&HCI, CPCI-S, CPCI-SSH, ESCI Timespan=All years
#3	TS=(cohort OR incidence OR survival OR prospective OR prospectively OR follow-up OR follow* up OR longitudinal) Indexes=SCI-EXPANDED, SSCI, A&HCI, CPCI-S, CPCI-SSH, ESCI Timespan=All years
#4	TS=(China OR Taiwan OR Japan OR Korea OR Mongolia OR Brunei OR Cambodia OR Indochina OR Indonesia OR Laos OR Malaysia OR Mekong Valley OR Myanmar OR Philippines OR Singapore OR Thailand OR Timor-Leste OR Vietnam OR Afghanistan OR Sri Lanka OR

	Bangladesh OR Bhutan OR India OR Nepal OR Pakistan OR Maldives)	Indexes=SCI-EXPANDED, SSCI, A&HCI, CPCI-S, CPCI-SSH, ESCI Timespan=All years
#5	#1 AND #2	
#6	#1 AND #2 AND #3	
#7	#1 AND #2 AND #3 AND #4	

Table S2. Risk of bias of the included studies: the Newcastle–Ottawa Scale.

Study	Selection of cohorts	Comparability of cohorts	Assessment of outcome	Total score
Zhang et al, 2021 [1]	3	2	2	7
Zhou et al, 2020 [2]	3	2	3	8
Zhou et al, 2022 [3]	3	2	3	8
Aihemaitijiang et al, 2022 [4]	3	2	2	7
Hata et al, 2022 [5]	4	2	3	9
Zhang et al, 2020 [6]	4	2	3	9
Matsuyama et al. 2019 [7]	4	2	3	9
Tomata et al, 2012 [8]	4	2	2	8
Chan et al, 2014 [9]	4	2	2	8
Pei et al, 2022 [10]	4	2	2	8
Matsuoka et al, 2017 [11]	3	2	3	8
Tsai et al, 2011 [12]	4	2	2	8
Fann et al, 2022 [13]	4	2	3	9
Zhang et al, 2022 [14]	3	2	3	8
Zhang et al, 2020 [15]	3	2	2	7
Qin et al, 2015 [16]	3	2	3	8
Shang et al, 2021 [17]	3	2	3	8
Xu et al, 2018 [18]	3	2	3	8
Zhang et al, 2023 [19]	3	2	3	8
Li et al, 2019 [20]	3	2	3	8
Qin et al, 2014 [21]	3	2	3	8
Gao et al, 2022 [22]	3	2	3	8
Sukik et al, 2022 [23]	3	2	3	8
Shi et al, 2019 [24]	3	2	3	8
Shi et al, 2019 [25]	3	2	3	8
Jiang et al, 2022 [26]	3	2	3	8
Zheng et al, 2021 [27]	4	2	2	8
Zhu et al, 2022 [28]	4	2	3	9
Wang et al, 2020 [29]	3	2	2	7
Hu et al, 2023 [30]	4	2	3	9
Chen et al, 2012 [31]	4	2	2	8
Wu et al, 2019 [32]	3	2	3	8
Tong et al, 2021 [33]	3	2	3	8
Sheng et al, 2021 [34]	3	2	3	8
Zhang et al, 2020 [35]	3	2	3	8
Jiang et al, 2020 [36]	3	2	3	8
Talaei et al, 2021 [37]	3	2	3	8
Sheng et al, 2020 [38]	3	2	3	8
Sheng et al, 2022 [39]	3	2	3	8
Jiang et al, 2021 [40]	3	2	3	8
Jiang et al, 2020 [41]	3	2	3	8
Ozawa et al, 2013 [42]	4	2	3	9
Kimura al, 2022 [43]	4	2	3	9
Ozawa et al, 2014 [44]	4	2	3	9
Ozawa et al, 2012 [45]	4	2	3	9

Otsuka et al, 2023 [46]	3	2	3	8
Murai et al, 2021 [47]	3	2	3	8
Svensson et al, 2022 [48]	3	2	3	8
Nozakia et al, 2021 [49]	3	2	3	8
Zhang et al, 2023 [50]	4	2	3	9
Kinoshita et al, 2021 [51]	4	2	3	9
Shirai, et al, 2019 [52]	4	2	3	9
Nakamoto et al, 2017 [53]	4	2	3	9
Tsurumaki et al, 2019 [54]	3	2	3	8
Tomata et al, 2016 [55]	3	2	2	7
Chou, et al, 2019 [56]	4	2	2	8
Li et al, 2022 [57]	4	2	3	9
Yeung, et al, 2022 [58]	4	2	2	8
Chuang et al, 2019 [59]	4	2	3	9
Lee et al, 2017 [60]	4	2	3	9
Chen et al, 2017 [61]	3	2	2	7
Tsai et al, 2014 [62]	3	2	3	8
Wang et al, 2022 [63]	4	2	3	9
Jia et al, 2023 [64]	4	2	3	9
Zhu et al, 2018 [65]	3	2	3	8
Liu et al, 2017 [66]	3	2	2	7
Gao et al, 2011 [67]	4	2	2	8
Manacharoen et al, 2023 [68]	4	2	3	9
Tao et al, 2019 [69]	4	2	2	8
Luo et al, 2022 [70]	4	2	3	9
Wang et al, 2021 [71]	3	2	2	7

References

1. Zhang, J.; Zhao, A. Dietary Diversity and Healthy Aging: A Prospective Study. *Nutrients* **2021**, *13*, doi:10.3390/nu13061787.
2. Zhou, Y.F.; Song, X.Y.; Wu, J.; Chen, G.C.; Neelakantan, N.; van Dam, R.M.; Feng, L.; Yuan, J.M.; Pan, A.; Koh, W.P. Association Between Dietary Patterns in Midlife and Healthy Ageing in Chinese Adults: The Singapore Chinese Health Study. *J Am Med Dir Assoc* **2021**, *22*, 1279-1286, doi:10.1016/j.jamda.2020.09.045.
3. Zhou, Y.F.; Lai, J.S.; Chong, M.F.; Tong, E.H.; Neelakantan, N.; Pan, A.; Koh, W.P. Association between changes in diet quality from mid-life to late-life and healthy ageing: the Singapore Chinese Health Study. *Age Ageing* **2022**, *51*, doi:10.1093/ageing/afac232.
4. Aihemaitijiang, S.; Zhang, L.; Ye, C.; Halimulati, M.; Huang, X.; Wang, R.; Zhang, Z. Long-Term High Dietary Diversity Maintains Good Physical Function in Chinese Elderly: A Cohort Study Based on CLHLS from 2011 to 2018. *Nutrients* **2022**, *14*, doi:10.3390/nu14091730.
5. Hata, T.; Seino, S.; Yokoyama, Y.; Narita, M.; Nishi, M.; Hida, A.; Shinkai, S.; Kitamura, A.; Fujiwara, Y. Interaction of Eating Status and Dietary Variety on Incident Functional Disability among Older Japanese Adults. *J Nutr Health Aging* **2022**, *26*, 698-705, doi:10.1007/s12603-022-1817-5.
6. Zhang, J.; Zhao, A.; Wu, W.; Ren, Z.X.; Yang, C.L.; Wang, P.Y.; Zhang, Y.M. Beneficial Effect of Dietary Diversity on the Risk of Disability in Activities of Daily Living in Adults: A Prospective Cohort Study. *Nutrients* **2020**, *12*.
7. Matsuyama, S.; Zhang, S.; Tomata, Y.; Abe, S.; Tanji, F.; Sugawara, Y.; Tsuji, I. Association between improved adherence to the Japanese diet and incident functional disability in older people: The Ohsaki Cohort 2006 Study. *Clin Nutr* **2020**, *39*, 2238-2245, doi:10.1016/j.clnu.2019.10.008.
8. Tomata, Y.; Kakizaki, M.; Nakaya, N.; Tsuboya, T.; Sone, T.; Kuriyama, S.; Hozawa, A.; Tsuji, I. Green tea consumption and the risk of incident functional disability in elderly Japanese: the Ohsaki Cohort 2006 Study. *Am J Clin Nutr* **2012**, *95*, 732-739, doi:10.3945/ajcn.111.023200.
9. Chan, R.; Chan, D.; Woo, J. A prospective cohort study to examine the association between dietary patterns and depressive symptoms in older Chinese people in Hong Kong. *PLoS One* **2014**, *9*, e105760, doi:10.1371/journal.pone.0105760.
10. Pei, Z.F.; Zhang, J.J.; Qin, W.Z.; Hu, F.F.; Zhao, Y.; Zhang, X.H.; Cong, X.X.; Liu, C.L.; Xu, L.Z. Association between Dietary Patterns and Depression in Chinese Older Adults: A Longitudinal Study Based on CLHLS. *Nutrients* **2022**, *14*.
11. Matsuoka, Y.J.; Sawada, N.; Mimura, M.; Shikimoto, R.; Nozaki, S.; Hamazaki, K.; Uchitomi, Y.; Tsugane, S. Dietary fish, n-3 polyunsaturated fatty acid consumption, and depression risk in Japan: a population-based prospective cohort study. *Transl Psychiatry* **2017**, *7*, e1242, doi:10.1038/tp.2017.206.
12. Tsai, A.C.; Chang, T.L.; Chi, S.H. Frequent consumption of vegetables predicts lower risk of depression in older Taiwanese - results of a prospective population-based study. *Public Health Nutr* **2012**, *15*, 1087-1092, doi:10.1017/s1368980011002977.
13. Fann, L.Y.; Huang, S.H.; Huang, Y.C.; Chen, C.F.; Sun, C.A.; Wang, B.L.; Chien, W.C.; Lu, C.H. The Synergetic Impact of Physical Activity and Fruit and Vegetable Consumption on the Risk of Depression in Taiwanese Adults. *Int. J. Environ. Res. Public Health* **2022**, *19*.
14. Zhang, T.; Jiang, G.J.; Li, F.D.; Gu, X.; Zhai, Y.J.; Xu, L.; Wu, M.N.; Shen, H.W.; Lin, J.F. Soy product consumption and the risk of major depressive disorder in older adults: Evidence from a cohort study. *Front. Psychiatry* **2022**, *13*.
15. Zhang, J.; Zhao, A.; Wu, W.; Yang, C.L.; Ren, Z.X.; Wang, M.C.; Wang, P.Y.; Zhang, Y.M. Dietary Diversity Is Associated With Memory Status in Chinese Adults: A Prospective Study. *Frontiers in Aging Neuroscience* **2020**, *12*.
16. Qin, B.; Adair, L.S.; Plassman, B.L.; Batis, C.; Edwards, L.J.; Popkin, B.M.; Mendez, M.A. Dietary

Patterns and Cognitive Decline Among Chinese Older Adults. *Epidemiology* **2015**, *26*, 758-768, doi:10.1097/ede.0000000000000338.
17. Shang, X.W.; Hodge, A.M.; Hill, E.; Zhu, Z.T.; He, M.G. Associations of Dietary Pattern and Sleep Duration with Cognitive Decline in Community-Dwelling Older Adults: A Seven-Year Follow-Up Cohort Study. *J. Alzheimers Dis.* **2021**, *82*, 1559-1571.
18. Xu, X.Y.; Parker, D.; Shi, Z.M.; Byles, J.; Hall, J.; Hickman, L. Dietary Pattern, Hypertension and Cognitive Function in an Older Population: 10-Year Longitudinal Survey. *Front. Public Health* **2018**, *6*.
19. Zhang, X.; Huang, F.; Zhang, J.; Wei, Y.; Bai, J.; Wang, H.; Jia, X. Association between Micronutrient-Related Dietary Pattern and Cognitive Function among Persons 55 Years and Older in China: A Longitudinal Study. *Nutrients* **2023**, *15*, doi:10.3390/nu15030481.
20. Li, M.; Shi, Z. A Prospective Association of Nut Consumption with Cognitive Function in Chinese Adults aged 55+ _ China Health and Nutrition Survey. *J Nutr Health Aging* **2019**, *23*, 211-216, doi:10.1007/s12603-018-1122-5.
21. Qin, B.; Plassman, B.L.; Edwards, L.J.; Popkin, B.M.; Adair, L.S.; Mendez, M.A. Fish intake is associated with slower cognitive decline in Chinese older adults. *J Nutr* **2014**, *144*, 1579-1585, doi:10.3945/jn.114.193854.
22. Gao, R.; Yang, Z.; Yan, W.; Du, W.; Zhou, Y.; Zhu, F. Protein intake from different sources and cognitive decline over 9 years in community-dwelling older adults. *Front Public Health* **2022**, *10*, 1016016.
23. Sukik, L.; Liu, J.H.; Shi, Z.M. Tea Consumption Is Associated with Reduced Cognitive Decline and Interacts with Iron Intake: A Population-Based Longitudinal Study on 4,820 Old Adults. *J. Alzheimers Dis.* **2022**, *90*, 271-282.
24. Shi, Z.M.; El-Obeid, T.; Riley, M.; Li, M.; Page, A.; Liu, J.H. High Chili Intake and Cognitive Function among 4582 Adults: An Open Cohort Study over 15 Years. *Nutrients* **2019**, *11*.
25. Shi, Z.M.; Li, M.; Wang, Y.F.; Liu, J.H.; El-Obeid, T. High iron intake is associated with poor cognition among Chinese old adults and varied by weight status-a 15-y longitudinal study in 4852 adults. *Am. J. Clin. Nutr.* **2019**, *109*, 109-116.
26. Jiang, K.; Xie, C.X.; Li, Z.R.; Zeng, H.; Zhao, Y.; Shi, Z.M. Selenium Intake and its Interaction with Iron Intake Are Associated with Cognitive Functions in Chinese Adults: A Longitudinal Study. *Nutrients* **2022**, *14*.
27. Zheng, J.Z.; Zhou, R.; Li, F.R.; Chen, L.R.; Wu, K.Y.; Huang, J.H.; Liu, H.M.; Huang, Z.W.; Xu, L.; Yuan, Z.L.; et al. Association between dietary diversity and cognitive impairment among the oldest-old: Findings from a nationwide cohort study. *Clinical Nutrition* **2021**, *40*, 1452-1462.
28. Zhu, A.N.; Yuan, C.Z.; Pretty, J.; Ji, J.S. Plant-based dietary patterns and cognitive function: A prospective cohort analysis of elderly individuals in China (2008-2018). *Brain Behav.* **2022**, *12*.
29. Wang, Z.; Pang, Y.; Liu, J.; Wang, J.; Xie, Z.; Huang, T. Association of healthy lifestyle with cognitive function among Chinese older adults. *Eur J Clin Nutr* **2021**, *75*, 325-334, doi:10.1038/s41430-020-00785-2.
30. Hu, W.; Zhang, H.; Ni, R.; Cao, Y.; Fang, W.; Chen, Y.; Pan, G. Interaction between the animal-based dietary pattern and green space on cognitive function among Chinese older adults: A prospective cohort study. *Int J Hyg Environ Health* **2023**, *250*, 114147.
31. Chen, X.; Huang, Y.; Cheng, H.G. Lower intake of vegetables and legumes associated with cognitive decline among illiterate elderly Chinese: a 3-year cohort study. *J Nutr Health Aging* **2012**, *16*, 549-552, doi:10.1007/s12603-012-0023-2.
32. Wu, J.; Song, X.Y.; Chen, G.C.; Neelakantan, N.; van Dam, R.M.; Feng, L.; Yuan, J.M.; Pan, A.; Koh, W.P. Dietary pattern in midlife and cognitive impairment in late life: a prospective study in Chinese adults. *Am. J. Clin. Nutr.* **2019**, *110*, 912-920.
33. Tong, E.H.; Lai, J.S.; Whitton, C.; Neelakantan, N.; Zhou, Y.F.; Chen, C.; van Dam, R.M.; Feng, L.; Pan, A.; Chong, M.F.F.; et al. Changes in Diet Quality from Mid- to Late Life Are Associated with Cognitive Impairment in the Singapore Chinese Health Study. *J. Nutr.* **2021**, *151*, 2800-

2807.
34. Sheng, L.T.; Jiang, Y.W.; Feng, L.; Pan, A.; Koh, W.P. Dietary Total Antioxidant Capacity and Late-Life Cognitive Impairment: The Singapore Chinese Health Study. *J Gerontol A Biol Sci Med Sci* **2022**, *77*, 561-569, doi:10.1093/gerona/glab100.
35. Zhang, Y.G.; Wu, J.; Feng, L.; Yuan, J.M.; Koh, E.P.; Pan, A. [Sugar-sweetened beverages consumption in midlife and risk of late-life cognitive impairment in Chinese adults]. *Zhonghua Liu Xing Bing Xue Za Zhi* **2020**, *41*, 55-61, doi:10.3760/cma.j.issn.0254-6450.2020.01.011.
36. Jiang, Y.W.; Sheng, L.T.; Pan, X.F.; Feng, L.; Yuan, J.M.; Pan, A.; Koh, W.P. Meat consumption in midlife and risk of cognitive impairment in old age: the Singapore Chinese Health Study. *Eur J Nutr* **2020**, *59*, 1729-1738, doi:10.1007/s00394-019-02031-3.
37. Talaei, M.; Feng, L.; Yuan, J.M.; Pan, A.; Koh, W.P. Dairy, soy, and calcium consumption and risk of cognitive impairment: the Singapore Chinese Health Study. *Eur J Nutr* **2020**, *59*, 1541-1552, doi:10.1007/s00394-019-02010-8.
38. Sheng, L.T.; Jiang, Y.W.; Pan, X.F.; Feng, L.; Yuan, J.M.; Pan, A.; Koh, W.P. Association Between Dietary Intakes of B Vitamins in Midlife and Cognitive Impairment in Late-Life: The Singapore Chinese Health Study. *J Gerontol A Biol Sci Med Sci* **2020**, *75*, 1222-1227, doi:10.1093/gerona/glz125.
39. Sheng, L.T.; Jiang, Y.W.; Alperet, D.J.; Feng, L.; Pan, A.; Koh, W.P. Quantity and variety of fruit and vegetable intake in midlife and cognitive impairment in late life: a prospective cohort study. *British Journal of Nutrition.* **2022**.
40. Jiang, Y.W.; Sheng, L.T.; Feng, L.; Pan, A.; Koh, W.P. Consumption of dietary nuts in midlife and risk of cognitive impairment in late-life: the Singapore Chinese Health Study. *Age and ageing.* **2020**, *18*.
41. Jiang, Y.W.; Sheng, L.T.; Pan, X.F.; Feng, L.; Yuan, J.M.; Pan, A.; Koh, W.P. Midlife Dietary Intakes of Monounsaturated Acids, n-6 Polyunsaturated Acids, and Plant-Based Fat Are Inversely Associated with Risk of Cognitive Impairment in Older Singapore Chinese Adults. *J. Nutr.* **2020**, *150*, 901-909.
42. Ozawa, M.; Ninomiya, T.; Ohara, T.; Doi, Y.; Uchida, K.; Shirota, T.; Yonemoto, K.; Kitazono, T.; Kiyohara, Y. Dietary patterns and risk of dementia in an elderly Japanese population: the Hisayama Study. *Am J Clin Nutr* **2013**, *97*, 1076-1082, doi:10.3945/ajcn.112.045575.
43. Kimura, Y.; Yoshida, D.; Ohara, T.; Hata, J.; Honda, T.; Hirakawa, Y.; Shibata, M.; Oishi, E.; Sakata, S.; Furuta, Y.; et al. Long-term association of vegetable and fruit intake with risk of dementia in Japanese older adults: the Hisayama study. *BMC Geriatr* **2022**, *22*, 257, doi:10.1186/s12877-022-02939-2.
44. Ozawa, M.; Ohara, T.; Ninomiya, T.; Hata, J.; Yoshida, D.; Mukai, N.; Nagata, M.; Uchida, K.; Shirota, T.; Kitazono, T.; et al. Milk and dairy consumption and risk of dementia in an elderly Japanese population: the Hisayama Study. *J Am Geriatr Soc* **2014**, *62*, 1224-1230, doi:10.1111/jgs.12887.
45. Ozawa, M.; Ninomiya, T.; Ohara, T.; Hirakawa, Y.; Doi, Y.; Hata, J.; Uchida, K.; Shirota, T.; Kitazono, T.; Kiyohara, Y. Self-reported dietary intake of potassium, calcium, and magnesium and risk of dementia in the Japanese: the Hisayama Study. *J Am Geriatr Soc* **2012**, *60*, 1515-1520, doi:10.1111/j.1532-5415.2012.04061.x.
46. Otsuka, R.; Zhang, S.; Ihira, H.; Sawada, N.; Inoue, M.; Yamagishi, K.; Yasuda, N.; Tsugane, S. Dietary diversity and risk of late-life disabling dementia in middle-aged and older adults. *Clinical Nutrition* **2023**, *42(4)*, 541-549.
47. Murai, U.; Sawada, N.; Charvat, H.; Inoue, M.; Yasuda, N.; Yamagishi, K.; Tsugane, S. Soy product intake and risk of incident disabling dementia: the JPHC Disabling Dementia Study. *Eur. J. Nutr.* **2022**, *61*, 4045-4057.
48. Svensson, T.; Sawada, N.; Mimura, M.; Nozaki, S.; Shikimoto, R.; Tsugane, S. Midlife intake of the isoflavone genistein and soy, and the risk of late-life cognitive impairment: the JPHC Saku Mental Health Study. *Journal of epidemiology.* **2021**, *18*.

49. Nozaki, S.; Sawada, N.; Matsuoka, Y.J.; Shikimoto, R.; Mimura, M.; Tsugane, S. Association Between Dietary Fish and PUFA Intake in Midlife and Dementia in Later Life: The JPHC Saku Mental Health Study. *J Alzheimers Dis* **2021**, *79*, 1091-1104, doi:10.3233/jad-191313.
50. Zhang, S.; Otsuka, R.; Nishita, Y.; Tange, C.; Tomida, M.; Ando, F.; Shimokata, H.; Arai, H. Twenty-year prospective cohort study of the association between a Japanese dietary pattern and incident dementia: the NILS-LSA project. *European Journal of Nutrition.* **2023**.
51. Kinoshita, K.; Otsuka, R.; Takada, M.; Tsukamoto-Yasui, M.; Nishita, Y.; Tange, C.; Tomida, M.; Shimokata, H.; Kuzuya, M.; Imaizumi, A.; et al. The Association between Dietary Amino Acid Intake and Cognitive Decline 8 Years Later in Japanese Community-Dwelling Older Adults. *J Nutr Health Aging* **2021**, *25*, 165-171, doi:10.1007/s12603-020-1470-9.
52. Shirai, Y.; Kuriki, K.; Otsuka, R.; Kato, Y.; Nishita, Y.; Tange, C.; Tomida, M.; Imai, T.; Ando, F.; Shimokata, H. Green tea and coffee intake and risk of cognitive decline in older adults: the National Institute for Longevity Sciences, Longitudinal Study of Aging. *Public Health Nutr* **2020**, *23*, 1049-1057, doi:10.1017/s1368980019002659.
53. Nakamoto, M.; Otsuka, R.; Nishita, Y.; Tange, C.; Tomida, M.; Kato, Y.; Imai, T.; Sakai, T.; Ando, F.; Shimokata, H. Soy food and isoflavone intake reduces the risk of cognitive impairment in elderly Japanese women. *Eur J Clin Nutr* **2018**, *72*, 1458-1462, doi:10.1038/s41430-017-0061-2.
54. Tsurumaki, N.; Zhang, S.; Tomata, Y.; Abe, S.; Sugawara, Y.; Matsuyama, S.; Tsuji, I. Fish consumption and risk of incident dementia in elderly Japanese: the Ohsaki cohort 2006 study. *Br J Nutr* **2019**, *122*, 1182-1191, doi:10.1017/s0007114519002265.
55. Tomata, Y.; Sugiyama, K.; Kaiho, Y.; Honkura, K.; Watanabe, T.; Zhang, S.; Sugawara, Y.; Tsuji, I. Dietary Patterns and Incident Dementia in Elderly Japanese: The Ohsaki Cohort 2006 Study. *J Gerontol A Biol Sci Med Sci* **2016**, *71*, 1322-1328, doi:10.1093/gerona/glw117.
56. Chou, Y.C.; Lee, M.S.; Chiou, J.M.; Chen, T.F.; Chen, Y.C.; Chen, J.H. Association of Diet Quality and Vegetable Variety with the Risk of Cognitive Decline in Chinese Older Adults. *Nutrients* **2019**, *11*, doi:10.3390/nu11071666.
57. Li, F.D.; Tong, Z.D.; Chang, Y.; Li, K.F.; Gu, X.; Zhang, T.; Lin, J.F. Eggs Consumption in Relation to Lower Risk of Cognitive Impairment in Elderly: Findings from a 6-Year Cohort Study. *J Nutr Health Aging* **2022**, *26*, 771-777, doi:10.1007/s12603-022-1810-z.
58. Yeung, S.S.Y.; Kwok, T.; Woo, J. Higher fruit and vegetable variety associated with lower risk of cognitive impairment in Chinese community-dwelling older men: a 4-year cohort study. *Eur J Nutr* **2022**, *61*, 1791-1799, doi:10.1007/s00394-021-02774-y.
59. Chuang, S.Y.; Lo, Y.L.; Wu, S.Y.; Wang, P.N.; Pan, W.H. Dietary Patterns and Foods Associated With Cognitive Function in Taiwanese Older Adults: The Cross-sectional and Longitudinal Studies. *J Am Med Dir Assoc* **2019**, *20*, 544-550 e544, doi:10.1016/j.jamda.2018.10.017.
60. Lee, A.T.C.; Richards, M.; Chan, W.C.; Chiu, H.F.K.; Lee, R.S.Y.; Lam, L.C.W. Lower risk of incident dementia among Chinese older adults having three servings of vegetables and two servings of fruits a day. *Age Ageing* **2017**, *46*, 773-779, doi:10.1093/ageing/afx018.
61. Chen, Y.C.; Jung, C.C.; Chen, J.H.; Chiou, J.M.; Chen, T.F.; Chen, Y.F.; Tang, S.C.; Yeh, S.J.; Lee, M.S. Association of Dietary Patterns With Global and Domain-Specific Cognitive Decline in Chinese Elderly. *J Am Geriatr Soc* **2017**, *65*, 1159-1167, doi:10.1111/jgs.14741.
62. Tsai, H.J. Dietary patterns and cognitive decline in Taiwanese aged 65 years and older. *Int J Geriatr Psychiatry* **2015**, *30*, 523-530, doi:10.1002/gps.4176.
63. Wang, R.S.; Wang, B.L.; Huang, Y.N.; Wan, T.T.H. The combined effect of physical activity and fruit and vegetable intake on decreasing cognitive decline in older Taiwanese adults. *Scientific Reports* **2022**, *12*.
64. Jia, J.; Zhao, T.; Liang, Y.; Liu, W.; Li, F.; Shi, S.; Zhou, C.; Yang, H.; Liao, Z.; Li, Y.; et al. Association between healthy lifestyle and memory decline in older adults: 10 year, population based, prospective cohort study. *BMJ* **2023**, *(no pagination)*.
65. Zhu, J.; Xiang, Y.B.; Cai, H.; Li, H.; Gao, Y.T.; Zheng, W.; Shu, X.O. A Prospective Investigation of Dietary Intake and Functional Impairments among the Elderly. *Am J Epidemiol* **2018**, *187(11)*,

2372-2386.
66. Liu, Z.M.; Tse, L.A.; Chen, B.; Wu, S.; Chan, D.; Kowk, T.; Woo, J.; Xiang, Y.T.; Wong, S.Y.S. Dietary acrylamide exposure was associated with mild cognition decline among non-smoking Chinese elderly men. *Sci Rep* **2017**, *7(1)*, 6395.
67. Gao, Q.; Niti, M.; Feng, L.; Yap, K.B.; Ng, T.P. Omega-3 Polyunsaturated Fatty Acid Supplements and Cognitive Decline: Singapore Longitudinal Aging Studies. *J. Nutr. Health Aging* **2011**, *15*, 32-35.
68. Manacharoen, A.; Jayanama, K.; Ruangritchankul, S.; Vathesatogkit, P.; Sritara, P.; Warodomwichit, D. Association of body mass index and dietary intake with mild cognitive impairment and dementia: a retrospective cohort study. *BMC Geriatr.* **2023**, *23*.
69. Tao, L.W.; Liu, K.; Chen, S.; Yu, H.Y.; An, Y.; Wang, Y.; Zhang, X.N.; Wang, Y.S.; Qin, Z.S.; Xiao, R. Dietary Intake of Riboflavin and Unsaturated Fatty Acid Can Improve the Multi-Domain Cognitive Function in Middle-Aged and Elderly Populations: A 2-Year Prospective Cohort Study. *Frontiers in Aging Neuroscience* **2019**, *11*.
70. Luo, J.; Zhang, C.; Zhao, Q.; Wu, W.; Liang, X.; Xiao, Z.; Mortimer, J.A.; Borenstein, A.R.; Dai, Q.; Ding, D. Dietary calcium and magnesium intake and risk for incident dementia: The Shanghai Aging Study. *Alzheimers Dement (N Y)* **2022**, *8*, e12362, doi:10.1002/trc2.12362.
71. Wang, L.J.; Liu, K.; Zhang, X.N.; Wang, Y.S.; Liu, W.; Wang, T.; Hao, L.; Ju, M.W.; Xiao, R. The Effect and Mechanism of Cholesterol and Vitamin B-12 on Multi-Domain Cognitive Function: A Prospective Study on Chinese Middle-Aged and Older Adults. *Frontiers in Aging Neuroscience* **2021**, *13*.
